# A Neighbor-Based Approach to Identify Tuberculosis Exposure, the Kopanyo Study 

**DOI:** 10.3201/eid2605.191568

**Published:** 2020-05

**Authors:** Patrick K. Moonan, Nicola M. Zetola, James L. Tobias, Joyce Basotli, Rosanna Boyd, Eleanor S. Click, Mbatshi Dima, Othusitse Fane, Alyssa M. Finlay, Matsiri Ogopotse, Xiao J. Wen, Chawangwa Modongo, John E. Oeltmann

**Affiliations:** Centers for Disease Control and Prevention, Atlanta, Georgia, USA (P.K. Moonan, R. Boyd, E.S. Click, A.M. Finlay, X.J. Wen, J.E. Oeltmann);; Botswana-UPenn Partnership, Gaborone, Botswana (N.M. Zetola, M. Dima, O. Fane, M. Ogopotse, C. Modongo);; Northrop Grumman, Atlanta (J.L. Tobias);; National Tuberculosis Program, Botswana Ministry of Health, Gaborone (J. Basotli)

**Keywords:** Mycobacterium tuberculosis, tuberculosis, TB, contact investigation, contact tracing, mapping, GIS, geographic information systems, nearest neighbor, neighborhoods, transmission, prevention, respiratory diseases, bacteria, Botswana, tuberculosis and other mycobacteria

## Abstract

Contact investigation is one public health measure used to prevent tuberculosis by identifying and treating persons exposed to *Mycobacterium tuberculosis*. Contact investigations are a major tenet of global tuberculosis elimination efforts, but for many reasons remain ineffective. We describe a novel neighbor-based approach to reframe contact investigations.

Tuberculosis (TB) is a global health emergency (*1*). The World Health Organization (WHO) End TB Strategy proposes a 90% reduction in TB incidence and 95% reduction in TB deaths by 2035 compared with 2015 (*2*). To reach this target, effective interventions are needed to interrupt transmission of *Mycobacterium tuberculosis*. Contact investigations help prevent *M. tuberculosis* transmission by identifying and treating persons in close contact with persons with TB disease (*3*). WHO recommends tuberculosis preventive treatment (TPT) for household members of bacteriologically confirmed pulmonary TB patients to prevent progression to active TB disease (*4*). 

Contact investigations are a major tenet of the End TB Strategy but remain ineffective for various reasons (*2*,*5*,*6*). Many TB programs in high-burden areas limit contact investigations to household members (*6*). Recent studies suggest that such restrictions might miss key exposures in the community (*7*,*8*). Targeted, population-based, geographic TB screening is a potential approach to augment contact investigations (*9*–*11*) but is resource and time intensive and rarely includes TPT (*11*,*12*). We used population-based, molecular epidemiologic data from Botswana to investigate potential use of a neighbor-based approach for contact investigations.

## The Study

During August 2012–April 2016, we enrolled participants treated for TB disease at 30 healthcare facilities in Botswana for a prospective molecular epidemiologic study, Kopanyo. In brief, Kopanyo was designed to explore potential clinical, demographic, geographic, social relationships, and *M. tuberculosis* genotypic characteristics among persons with TB (*13*,*14*). We interviewed enrolled patients by using a standardized questionnaire and abstracted clinical data from medical records (*13*). We collected and processed sputum samples for culture and genotyped isolates with 24-locus mycobacterial interspersed repetitive units–variable-number tandem-repeats by using standard methods (*15*). We geocoded and validated the primary residence of each enrolled patient (Appendix). We excluded patients without a validated primary residential geocode and those who resided in locations outside of the study area. The study area included all 11 neighborhoods in Gaborone and 3 villages in the Ghanzi District: Ghanzi, D’Kar, and Kuke.

We defined index patients as the first culture-positive pulmonary TB patient identified and started on treatment in a household. We used residence plots to identify nearest neighbors, which we defined as those who lived immediately next door, and next-nearest neighbors, which we defined as those who lived 2 doors away (Figure). We enumerated all subsequent TB cases identified by bacteriologic confirmation and clinical diagnosis within the index home, nearest-neighbor homes, and next-nearest neighbor homes. We defined future-related patients as culture-positive patients with matching genotypes diagnosed after exposure to an index patient. Concurrent disease was TB diagnosed in a contact within 90 days of the index patient. 

**Figure:**
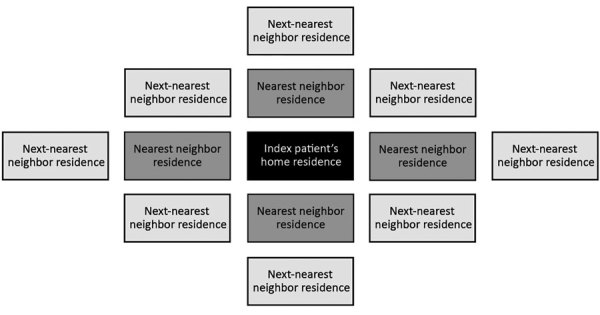
Illustration of possible nearest neighbors and next-nearest neighbors for tuberculosis (TB) screening and possible TB preventive treatment. Black box represents the home of a TB index patient; dark-gray boxes represent the nearest-neighbor homes; light-gray boxes represent the next-nearest neighbor homes. This figure does not reflect the true number of neighbor homes, and index patients might have >4 next-door neighbors, depending on the geographic orientation of residential plots.

We enrolled 4,331 patients but excluded 595 (14%) without a residential geocode and 547 (13%) who resided outside the study area. We analyzed data on the remaining 3,189 patients. Among 1,072 index patients, 143 (13%) had subsequent TB patients in the home (n = 426); 30 (7%) in-home subsequent patients had concurrent disease. Of 1,072 index patients, 73 (7%) had future-related patients (n = 123) in their homes; 5 (3.94%) of those had concurrent TB disease.

 When we applied a neighbor-based approach, we noted that 257 (24%) index patients could have subsequent TB patients living next door (n = 749), 41 of which could have concurrent disease. Among next-nearest neighbors of index patients, 390 (36%) could have subsequent TB, 23 of which could have concurrent disease (Table). In addition, 29 (2.7%) index patients could have future-related patients among their nearest neighbors (n = 42), and 5 (0.5%) future-related patients among next-nearest neighbors (n = 10), 3 with concurrent TB disease (Table).

**Table:** Number of index patients and possible additional subsequent contacts and future-related patients identified by using a nearest-neighbor approach to tuberculosis screening, Botswana*

Geographic area	No. index patients†		No. household members (FR)‡	No. nearest-neighbors (FR)‡	No. next-nearest neighbors (FR)‡	Total subsequent patients (FR)‡	No. screened to identify 1 TB patient (95% CI)§	Household contacts that could benefit from TPT¶	Neighbor contacts that could benefit from TPT¶
Gaborone									
A	123		57 (16)	93 (0)	47 (2)	197 (18)	21 (13–32)	861	3,472
B	58		19 (4)	41 (0)	21 (0)	81 (4)	18 (11–28)	307	1,230
C	210		83 (22)	146 (8)	84 (1)	313 (31)	16 (9–26)	1,092	4,368
D	195		58 (10)	110 (0)	56 (2)	224 (12)	19 (11–30)	878	3,510
E	79		28 (6)	46 (0)	30 (0)	104 (6)	11 (5–20)	253	1,011
F	129		53 (2)	84 (2)	51 (2)	188 (6)	15 (8–25)	593	2,374
G	51		14 (0)	29 (0)	18 (0)	61 (0)	9 (4–17)	128	510
H	20		5 (0)	12 (0)	6 (0)	23 (0)	7 (3–14)	38	152
I	6		2 (0)	9 (0)	4 (0)	15 (0)	2 (0–7)	10	41
J	6		2 (0)	2 (0)	1 (0)	5 (0)	22 (14–33)	23	94
K	11		6 (0)	11 (0)	6 (0)	23 (0)	7 (3–14)	35	141
Ghanzi District									
Ghanzi	141		83 (57)	143 (24)	57 (3)	283 (84)	6 (2–16)	398	1,590
D’kar	35		9 (2)	14 (8)	7 (0)	30 (10)	11 (5–20)	86	280
Kuke	8		7 (4)	9 (0)	2 (0)	18 (4)	8 (3–15)	28	128
Total	1,072		426 (123)	749 (42)	390 (10)	1,565 (175)	16 (9–26)	4,730	18,901

*FR, future related; TB, tuberculosis; TPT, tuberculosis preventive treatment. †No. index patients is equivalent to the number of standard contact investigations. ‡Future related, i.e., all culture-positive patients with matching *M. tuberculosis* genotype as an index patient.  §Limits of 95% CI assume a Poisson distribution. ¶Number exposed to bacteriologically confirmed pulmonary TB who do not have TB disease.

We found that a neighbor-based approach could identify 1,565 additional subsequent TB patients, including 175 future-related patients, and 102 patients with concurrent TB disease. The number of persons living with a bacteriologically positive patient varied by geography; however, ≈23,630 contacts potentially could benefit from TPT. Of note, 9% (97/1,072) of index patients interviewed stated they lived alone, but 91 (94%) had subsequent patients identified in the home, and 84 (87%) had subsequent future-related patients living in the home.

## Conclusions

We explored the use of a nearest-neighbor approach to expand TB contact investigations. This approach does not rely on name-based contact identification, which has been shown to be ineffective (*6*,*16**–**18*). In addition, the neighbor-based approach would not require mobile screening units or mass screening campaigns in the community. By simply expanding the number of homes visited to nearest and next-nearest neighbors, the Botswana National TB Program could increase the number of TB case diagnoses by 146% and potentially interrupt 175 secondary patient transmission events.

Preventing future TB disease through TPT could also hasten TB elimination in at-risk neighborhoods and reduce deaths in the community (*11*,*12*). Cegielski et al. effectively used TPT to eliminate TB from 2 at-risk neighborhoods in Texas, USA (*11*). The focus on nearest and next-nearest neighbors gives programs a tangible and practical approach to locating persons at risk for TB exposure and progression to TB disease. 

The neighbor-based approach differs from a neighborhood screening, which places an additional burden on TB programs by unnecessarily screening many persons at lower risk. For example, 59,100 persons reside in neighborhood C in Gaborone (data not shown). Under the neighbor-based approach, only 5,470 (9%) persons, including in-home and nearest neighbor residents, would be targeted for testing. 

Previous reports suggest that contact investigations fail to identify key relationships, even within households (*16*,*17*). Potential stigma and lack of trust in government officials also play a role in contact investigations (*16*–*18*). In our cohort, many (n = 97) index patients said they lived alone, but 94% of them had subsequent cases identified in the home. In addition, 48% of future-related patients were linked to index patients who claimed no household contacts during name-based contact solicitation interviews conducted at the enrollment clinic. Household membership composition could have changed over time, and some connections might not have existed at the time of the interview. However, our study reinforces the necessity of home visits at times convenient to the index patient and when most household members are in the home, which might warrant home visits outside of business hours and flexibility in staff workplans.

Our analysis emphasizes the opportunity to prevent future TB patients and future-related TB patients by providing TPT. Household contacts, especially young children and persons living with HIV, are eligible for TPT by national policy, but TPT has not been practiced routinely in Botswana. As the Botswana Ministry of Health scales up access to TPT throughout the country, the neighbor-based approach could improve identification of most likely contacts and help target interventions where they are most needed.

Our study has limitations. Living in proximity to an index patient is not the only opportunity for transmission and might not always translate into time spent together. In addition, our analysis of future-related patients included only patients with culture-positive disease and genotyping results; excluding them did not affect the main analysis enumerating subsequent patients but might have underestimated the number of future-related patients. Also, our estimates for TPT represent the maximum number of persons who could benefit because we used the average number of persons per household and assumed all household members would be eligible for TPT without a reliable and available test for infection.

A neighbor-based approach should not supplant household investigations, and community-based interventions should not divert essential resources from those already devoted to finding and treating TB patients. Wide-scale implementation of this approach would require adequate resources to ensure that all patients complete the full cascade of treatment. To reach the ambitious global goal of TB elimination, we need simple, easy to implement, location-based approaches. Screening index patient households and nearest neighbors could help identify additional TB patients and persons who could benefit from TPT.

Appendix Additional information on a neighbor-based approach to identifying tuberculosis exposure, the Kopanyo Study.
