# Supplementary material for: A Neighbor-Based Approach to Identify Tuberculosis Exposure, the Kopanyo Study
Source: Emerg Infect Dis. 2020 May;26(5):1010–3. doi: 10.3201/eid2605.191568 (PMC7181937; doi:10.3201/eid2605.191568)
Supplement: Appendix — Additional information on a neighbor-based approach to identifying tuberculosis exposure, the Kopanyo Study. [file 19-1568-Techapp-s1.pdf]

Article DOI: https://doi.org/10.3201/eid2605.191568

A Neighbor-Based Approach to Identify Tuberculosis Exposure, the Kopanyo Study

Appendix

Additional Methods

We obtained valid 24-locus mycobacterial interspersed repetitive units–variable-number tandem-repeats genotyping results from 2,012 patient isolates. Patient primary residential address was obtained during face-to-face interview. All addresses were verified by site visit geotagging, or through a reference layer created by manually relocating addresses in satellite imagery (*1*) by using OpenStreetMap (http://www.openstreetmap.org), Google Maps (https://maps.google.com), and ArcGIS 10.0 (Environmental System Research Institute [ESRI], https://www.esri.com) online geocoding services. We exported World Geodetic System 84 (WGS 84; https://gisgeography.com) projection system latitude and longitude coordinates with 1.1-meter precision for each address.

We obtained records on residential parcels from the Botswana Survey and Lands Department for the cities of Gaborone and Ghanzi. Botswana Land Survey Act required that each parcel of land for cadastral surveys in both urban and rural areas were complete and tied to a common spatial reference. The parcels were delineated by traditional ground-based survey methods but also aligned orthographic photos from overhead flights and imagery data (*2*). An integrated Geographic Information System (GIS) project brought all cadastral, geodetic, and topographic databases together into a single Geodatabase (Oracle with ArcSDE). Shapefiles of the parcels were provided to study staff via formal government request. For the rural villages of D’Kar and Kuke, building footprints were digitized by manually relocating addresses in satellite imagery using OpenStreetMap. These footprints were used as a proxy for the official government parcels to approximate homesteads.

We defined neighborhood boundaries to enumerate localized populations necessary for TB incidence rates, and to enumerate the number of homes (parcels), and number of contacts per

house based on neighborhood-level population estimates (*3*). An average number of household members was calculated for each geographic location based on the number of households and population estimate of each area. According to international guidelines, all household members, regardless of age, exposed to bacteriologically confirmed pulmonary TB, who are not TB-positive are eligible for tuberculosis preventive treatment (TPT), regardless of availability of testing for latent infection (*4*).

Ethics Approval

This study was approved by the US Centers for Disease Control and Prevention Institutional Review Board (IRB no. 6291); Health Research and Development Committee, Ministry of Health and Wellness, Botswana (reference no. 13/18/1/891); and the University of Pennsylvania IRBs (protocol no. 815718). All enrolled participants provided written informed consent.

References

1. Faure E, Danjou AM, Clavel-Chapelon F, Boutron-Ruault MC, Dossus L, Fervers B. Accuracy of two geocoding methods for geographic information system-based exposure assessment in epidemiological studies. Environ Health. 2017;16:15. PubMed https://doi.org/10.1186/s12940-017-0217-5

2. Morebodi BBH. Botswana–Department of Surveys and Mapping (DSM) Cadastral Information System. 2006 Mar 13 [cited 2020 Mar 22]. https://www.fig.net/resources/proceedings/fig_proceedings/accra/papers/ts14/ts14_05_morebodi.pdf

3. Statistics Botswana. Population and housing census 2011: analytical report. Gaborone (Botswana); Statistics Botswana: 2014. [cited 2020 Mar 22] http://www.statsbots.org.bw/sites/default/files/publications/Population%20%26%20Housing%20Census%20Dissemination%20analytical%20report%20.pdf

4. World Health Organization. Latent tuberculosis infection: updated and consolidated guidelines for programmatic management. Geneva: The Organization; 2018.
